# Reviewing clinical guideline development tools: features and characteristics

**DOI:** 10.1186/s12911-017-0530-5

**Published:** 2017-09-04

**Authors:** Soudabeh Khodambashi, Øystein Nytrø

**Affiliations:** Department of computer and Information Science, Norwegian University of Science and Technology, Trondheim, Norway

**Keywords:** Clinical guideline, Guideline development tool, Electronic guideline authoring, Guideline development process

## Abstract

**Background:**

To improve consistency and streamline development and publication of clinical guidelines (GL), there is a need for appropriate software support. We have found few specific tools for the actual authoring and maintaining of GLs, and correspondingly few analyses or reviews of GL development tool functionality. In order to assist GL developers in selecting and evaluating tools, this study tries to address the perceived gap by pursuing four goals: 1) identifying available tools, 2) reviewing a representative group of tools and their supported functionalities, 3) uncovering themes of features that the studied tools support, and 4) compare the selected tools with respect to the themes.

**Methods:**

We conducted a literature search using PubMed and Google Scholar in order to find GL development tools (GDT). We also explored tools and Content Management Systems (CMS) used in representative organisations and international communities that develop and maintain GLs. By reading a selected representative group of five GL tool manuals, exploring tools hands-on, we uncovered 8 themes of features. All found tools were compared according to these themes in order to identify the level of functionality they offer to support the GL development and publishing process. In order to limit the scope, tools for designing computer-interpretable/executable GL are excluded.

**Results:**

After finding 1552 published papers, contacting 7 organizations and international communities, we identified a total of 19 unique tools, of which 5 tools were selected as representative in this paper. We uncovered a total of 8 themes of features according to the identified functionalities that each tool provides. Four features were common among tools: Collaborative authoring process support, user access control, GL repository management, electronic publishing. We found that the GRADE methodology was supported by three of the reviewed tools, while only two tools support annotating GL with MeSH terms. We also identified that monitoring progress, reference management, Managing versions (version control), and Change control (tracking) were often the missing features.

**Conclusion:**

The results can promote sector discussion and eventual agreement on important tool functionality. It may aid tool and GL developers towards more efficient, and effective, GL authoring.

**Electronic supplementary material:**

The online version of this article (10.1186/s12911-017-0530-5) contains supplementary material, which is available to authorized users.

## Background

Clinical practice guidelines (GL) are developed to provide evidence-based advice on diagnosis and treatment to clinicians at the point of care [1]. During the last two decades, major advances have been made in developing, disseminating and implementing GLs to improve healthcare outcomes [2]. Various guidelines on how to develop guidelines have been published, and standards are suggested for the development of trustworthy guidelines (guideline for guidelines) [3, 4]. The process of authoring GLs is complex, time consuming, and usually involves large, often geographically distributed, multidisciplinary teams. It is common to develop a guideline as an academic text, by means of a document editor, and most often the final version, with summary recommendations, is published in print and guideline web portals and services.

Organizations responsible for developing and maintaining GLs adopt Content Management Systems (CMS) in order to manage concurrent authoring, revision, review and publishing. Our previous case study on information systems support for maintaining national GLs in Norway showed that the currently used information systems or CMS are neither fulfilling the authors’ needs in terms of functionality, nor facilitating communication between authors developing guidelines collaboratively [5]. Different groups of researchers have proposed specialized software tools to ease the guideline development process while accommodating guideline authors’ needs. Collectively, we describe these tools as ‘guideline development tools’ (GDT) [6–9].

Developers of GDTs have their own assumptions and points of views, which results in software with different functionalities and features. In addition, the proposed GDTs have different foci in the guideline development process. To the best of our knowledge, there is no peer-reviewed paper analysing or comparing GDTs in detail. Therefore, characterising features supported by the GDTs, comparing them and presenting their area of focus were the main motivations behind this research. Consequently, this study has four main goals: 1) identifying available GDTs, 2) reviewing a representative group of GDTs and their supported functionalities, 3) uncovering themes of features that the studied GDTs support, and 4) compare the selected tools with respect to the themes.

We note that the scope of this study is limited to the GDTs for authoring GLs; therefore tools for guideline adaption and implementation are omitted. Furthermore, tools for authoring formalized (computer-interpretable) guidelines are also omitted, since they represent a distinct later step in GL development, overlapping with general software design and development tools that are well described in the research literature.

The rest of the article is organized as follows: The ‘Method’ section presents the search strategy to find GDTs and related CMSs, tool selection criteria, review method, and methods for uncovering themes. The ‘Results’ section presents the found GDTs, selected representative GDTs and themes of features. ‘Discussion’ tries to learn from the results, while ‘Conclusion’ acknowledges that tools must be tested in practice.

## Methods

### Searching for guideline development tools

We searched for peer-reviewed papers outlining relevant GDT or CMS in literature, contacted representative guideline development organisations and communities, and attended an international conference and a national meeting.

**Fig. 1:**
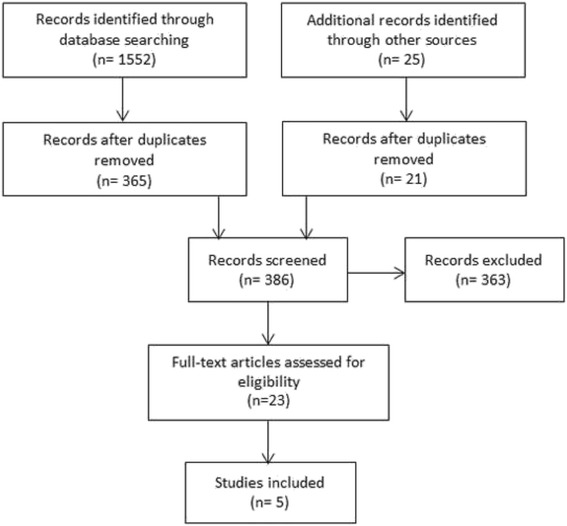
Selection process for literature about guideline development tools

The primary literature search was performed using PubMed and Google Scholar in March 2015 and the last search was conducted in May 2016. The selection and review process was conducted according to PRISMA guidelines [10] and is presented in Fig. 1. Details of retrieved papers based on a combination of search keywords are presented in Table 1.

**Table 1:** Search keywords and results of the literature review

Search phrases and keywords	Google Scholar		PubMed	
	No. retrieved papers	No. relevant papers	No. retrieved papers	No. relevant papers
“content management system” “clinical guideline”	21	0	95	1 [19]
“content management systems” “clinical guideline”	7	0	97	0
“content management systems” “clinical guidelines”	39	2 [19]	139	0
“content management system” “clinical guidelines”	91	2 [19]	149	1 [19]
“content management system” “guideline” “clinical”	282	2 [19]	95	1 [19]
“content management system” “guideline” “clinical” “CMS”	123	2 [19]	1	0
“guideline development tool” “guideline development”	56	4 [6, 14, 16, 17]	108	0
“guideline authoring tool” “guideline development”	17	1 [13]	6	0
“guideline authoring tools” “guideline development”	13	2 [13, 14]	12	2 [13, 19]
“guideline development” “software” “authoring tool”	56	3 [13, 15, 21]	2	0
“Development tool” and “clinical guideline”	69	1 [18]	28	0
“Integrated Development Environment” and “clinical recommendation”	1	0	0	0
“Integrated Development Environment” and “clinical guideline”	20	0	25	0

We reviewed the title and abstract of the retrieved papers. In case the title and abstract of the reviewed papers were not clear, we screened the full text. The two authors performed study selection in duplicate and discussed the inclusion or exclusion of papers until consensus was achieved.

In order to learn more about actual GDT in use, we contacted a sample of organizations and national/international communities with known GDT-related activity.

- ▪ Guideline International Network (G-I-N) [11].
- ▪ NICE (National Institute For Health and Clinical Excellence), United Kingdom
- ▪ Agency for Healthcare Research and Quality (AHRQ), USA
- ▪ National Health and Medical Research Council (NHMRC), Australia
- ▪ Norwegian Health Library (Helsebiblioteket)
- ▪ St. Olav’s University Hospital Trust (Norway)
- ▪ Innlandet Hospital Trust (Norway)

In addition, participation in two conferences was another source of identifying GDTs:

- ▪ DECIDE International Conference (http://www.cebhc.co.za/decide-conference), in Edinburgh, 2014, conference theme: evidence-based guideline development.
- ▪ The Norwegian Directorate of Health supplier conference, in Oslo, 2014, conference theme: procurement of potential GDT for authoring national clinical guidelines.

### The selection criteria of GDTs and CMSs for analysis

We considered three main criteria:

1. Some tools offer only partial support of the GL authoring process. In order to select a representative set of tools for comparison, we systematically ranked more general tools, covering more steps in the authoring process, higher than tools covering fewer steps. In the end, we omitted GDTs that were fully subsumed by more comprehensive tools.
2. General-purpose CMS were not considered for review, only CMSs specifically supporting GL authoring.
3. We excluded tools that only covered GL adaption, localization and/or implementation. GL or evidence profile repositories were excluded.

### Method for analysing the GDTs and CMS

We explored the GDTs and CMSs that we found in literature and organisations hands-on. We also analysed the provided manuals for each tool in order to identify the functionalities and features they support. If we questioned certain functionalities, we contacted the developing team directly.

### Method for uncovering themes

We uncovered themes of supported features and functionalities using the thematic synthesis method [12]. It allowed us to identify specific segments of text and recurring topics, label them, elicit high-level requirements and classify them into themes. Prose text descriptions were validated in seven brainstorming sessions between authors.

## Results

The results according to the goals are: 1) identified GDTs and CMSs, 2) a selection of representative GDT, 3) a set of feature themes, and 4) a characterization of the selected tools according to the identified themes.

### The identified GDTs and CMSs

The identified tools based on the searched sources are presented in the following sections.

#### Details of the literature search

Our literature search phrases and corresponding results were presented in Table 1. We generally found that publications describe the individual tools and their use, rather than their functionalities. In Table 1, MAGICapp is the subject of [13–15], GRADEPro is the subject of [16–18], and Internet Portal for guideline development (henceforth “Internet Portal”) is presented in [19, 20]. All the tools and methodologies reviewed by M. Peleg [21], except BRIDGE-Wiz [7], were aimed at computational representation, and require users to have programming knowledge or collaborate with knowledge engineers to encode guidelines; therefore, they are excluded from our study.

#### Details of search in organizations

The G-I-N have published a list of suggested tools on their website [22]. Again, we found MAGICapp, GRADEPro, and Internet Portal. The other suggested tools on the Website [22] are designed to support only part of the guideline development process. We categorise them below:

1. *Adapt and implement existing guidelines*: CAN-IMPLEMENT© [23]
2. *Make and publish summary of finding tables* (SoF): DECIDE [24]
3. *Perform systematic review*: Doctor Evidence platforms [25], RevMan 5 [26], Distiller [27], Rayyan [28], JBI-SUMARI [29], SRDR [30], EPPI-Reviewer 4 [31], CREBP SRA Systematic Review Creator [32] and Covidence [33]
4. *Maintain systematic review repository:* Epistemonikos [34], and SRDR [30]
5. *Do semi-automated citation screening for systematic reviews*: Abstrackr [35]
6. *Maintain guideline repository*: GIN guideline library [36]
7. *Detect duplicates in systematic reviews*: CREBP Systematic Review Assistant [37]
8. *Maintain evidence profile repository*: GDTs database of evidence profiles [38]

By contacting to the other organizations, we found that NICE and NHMRC have their own in-house CMS; AHRQ does not use any tools or CMS. We found that the Norwegian Health Library, the St. Olav’s University Hospital Trust and the Innlandet Hospital Trust all used general-purpose CMS. The Norwegian Health Library piloted MAGICapp, and Innlandet Hospital Trust piloted purpose-built “Håndboka”.

#### Details of search in national/international communities

The Decide conference and the supplier conference uncovered, again, MAGICapp [15] and GRADEpro [39]. The national supplier conference furthermore uncovered Håndboka.

### Selected tools for review

According to the tool selection criteria presented in Section 2.2, the selected tools were: MAGICapp, GRADEpro, Internet Portal, BRIDGE-Wiz, and Håndboka. BRIDGE-Wiz [7] is designed to be used in the panel meeting to systematically create more transparent recommendations. BRIDGE-Wiz enhances implementation by letting authors state the strength of the recommendation, the level of obligation, and the balance between the benefits and harms [7]. Since the implemented features in BRIDGE-Wiz are different from other GDTs that fully support the GL development process, we included it in our analysis. The next section describes themes of features uncovered in these five GDTs.

### The uncovered themes of features

Based on hands-on experience and the thematic analysis method, we identified a total of 8 themes representing GDTs features: 1) Team and contribution management, 2) Project management, 3) Evidence management, 4) Guideline development, 5) Document management, 6) Guideline content enhancement, 7) Import, export and publication, 8) User experience enhancement. A list of identified themes, their description and intension are presented in Table 2.

**Table 2:** The identified themes and their features

Themes	Features	Description
Team and contribution management	Collaboration	Support guideline panel roles; Remote and synchronous/asynchronous collaboration; Separate development, review, approval, etc. Allow both central real-time collaboration and remote batch-wise check-in/out.
	Access	Enable logging; Authentication and authorization; Access policies and profiles; High granularity of access control; Separate between edit, comment, create, delete, review, vote, accept and other forms of access.
	Role	Support multiple roles per individual; Group roles (more than one person filling a role): Organizational roles; Dynamic roles according to process part and progress; Role inheritance and composition; Role-based access control; Dynamic roles according to voting and role delegation.
	Conflict of interest	Handle and track panel members COI declarations [47]. Accept different organizational COIs and content, for example: ICMJE [48], WHO [47], NHF (McMaster-NHF Guideline Panel conflict of interest form), and MSPSC [18].
Project management	Development checklist	Support development process phases, including evidence review, development, publication, evaluation, etc.; Support plans and plan templates; Progress monitoring and dashboards; Management of workload, work division and subprojects; Quality control and testing/validation; Cfr. Suggested lists by Guideline International Network [49], AGREE [50], and GIN-McMaster Guideline Development Checklist [51].
	Milestones and deadlines	Support for resource control and allocation, deliverable management
	Progress monitoring	Review and revise and monitor steps/phases and degree of completion. Both for participants, audits and management.
Evidence management	Search strategy and history	Support search in literature, existing guidelines and systematic reviews and health technology assessment reports for latest evidence according to a strategy [52]. Record search strategy, sources, search keywords, retrieved results, filtering, inclusion and exclusion criteria and outcome.
	Evidence repository	Support storage of annotated literature and summaries in context; Provide sharing and collaborative assessment.
	Citations and reference	Record evidence background for a specific recommendation. Link to context (cite) and refer to bibliography in evidence repository. Add, revise and delete background with justifications and assessment. Automate reference embedding and corresponding repository update.
Guideline development	Evidence assessment	Enable repeated assessment of articles and evidence; Document assessment including temporary and final decisions of relevance, currency and eligibility for inclusion. Support different methods for assessment rating, i.e. Scottish Intercollegiate Guidelines Network (SIGN) [53], Oxford [54], and GRADE [55].
	Quality rating	Support different methodologies for rating the quality of evidence and ranking the strength of recommendations [56]. i.e. Grades of Recommendation Assessment, Development and Evaluation (GRADE) [57, 58], the American Academy of Paediatrics (AAP), American College of Emergency Physicians (ACEP), American Society of Clinical Oncology (ASCO), American Urological Association (AUA), American Society for Parenteral and Enteral Nutrition (ASPEN), and American Physical Therapy Association (APTA) [7, 56, 59].
	Terminology and language	Support controlled natural language restricting the grammar and vocabulary in order to reduce or eliminate ambiguity and complexity [60]. Support limited sentence and recommendation forms to enhance consistency of expression. Deontic expressions (‘must’, ‘should’, ‘may’) can be used to add a semantics of intended level of obligation in the recommendation [7].
	Voting	Support voting process, including voting rules, collecting casted votes, results presentations and recording. Record justifications and factors like evidence behind the recommendations, values, preferences and available resources in approving the recommendations. Document disagreement and consensus processes [52].
Document management	Change control (tracking)	Since guideline development is a collaborative, complex editorial and review process, it is desirable to keep track of all the changes in the guideline content based on the collaborators user account in a distinguishable and comprehensible way. All the modification on guideline (partially or fully) including metadata associated to the guideline should be trackable.
	Managing versions (version control)	Since the guideline development process needs constant edition and update, successive iterations of a document have to be numbered and saved in the repository. The versioning control for a document library can include major versions and minor (draft) versions for the same guideline under development.
	Template-based authoring	To ensure consistency, template-based authoring can assist authors by indicating the main subject headings that are necessary to be included in a guideline. For example, a guideline has to have title, background information, recommendation and references. Different organisations have different perspective on the main components that should be included in a guideline. Therefore, template-based authoring with certain flexibility in addressing different organizational perspectives for different types of guidelines is required.
	Guideline repository management	The guideline development process is a nonlinear process. A guideline has to be changed frequently before final approval for publication. Therefore, storing the guidelines under development, which is a living document, in a repository facilitates the development process significantly. It can include storing images and all related attachments to the guideline as well.
Guideline content enhancement	Tagging and EHR linking	Support semi-automated tagging of recommendations with relevant classification as ICD [61], SNOMED-CT [62], ATC [63], RxNorm [64], MeSH [65], ICPC-2 [66], LOINC [67], and UMLS [68]. Support tagging content that may be retrieved from the EHR.
Import, export and publication	Import and export file formats	Guidelines, templates, meta-data, roles, repositories, process data (fully or partially) to different templates and formats. Between projects, guidelines and tools.
	Publishing	Export navigable and end-usable guidelines
User experience enhancement	Wizard-based authoring	Sequencing of steps in the guideline development process (fully or partially) in order to lead the authors through a series of well-defined steps.
	Walkthrough user guide	A walkthrough user guide to provide a step-by-step overview of implemented functionalities in a guideline development tool to the users with no experience
	User manual	Software user guide containing details on how to navigate and use the implemented functionalities.

### Representative tools characterization

We reviewed the five representative tools (MAGICapp, GRADEpro, BRIDGE-Wiz, Håndboka, and Internet Portal) based on the uncovered themes and present the results in in Table 3. In case the functionality/feature is not supported by the tool, we used ‘-’ sign. Details of the results are presented in the see Additional file 1.

**Table 3:** Support of the selected tools with respect to themes and their features

Theme	Features	MAGICapp	GRADEpro	BRIDGE-Wiz	Håndboka	Internet Portal
Team and contribution management	collaboration	Web-based application	Web-based application	-	Web-based application	Web-based application
	Access	• Sign-up as a new user • Sign in with Google+	Sign-up as a new user	-	By invitation to email address	By invitation to email address
	Role	• Viewer • Reviewer • Author • Admin	• Admin • Researcher • No access • Panel member	-	-	• Coordinator • Leader • Members • Patient • Expert
	Conflict of interest	-	Based on: • ICMJE [48] • WHO [47] • NHF • MSPSC [18]	-	-	Their own COI form
Project management	Development checklist	• Planning • Content development • Publication, evaluation and updating	-	-	-	-
	Milestones and deadlines	• Task title • Date • Owner • Comment	• Task title • Due date	-	-	• Title • Date • Time • Place • Contact person
	Progress monitoring	Manual • Evidence profiles • Guidelines	-	-	-	• Voting status • survey status • Activity log
Evidence management	Search strategy and history	Redirect search query to 6 external search engines: -Epistemonikos [69] -SuperFilters -PubMed -International Guideline Library -Dynamed -The Knowledge Egg	-	-	-	Document search strategy along with the import history in a literature collection.
	Evidence repository	As an attached file to an imported reference	-	-	-	• Bibliographic data • Abstract • Attach files (PDF)
	Citations	From reference library (imported references)	By adding hyperlink	-	By adding hyperlink	Link the citation manually
	Reference manager	To some extent	-	-	-	-
Guideline development	Evidence assessment	GRADE	GRADE	-	-	• Sign [53] • Oxford [54]
	Quality rating	GRADE	GRADE	• GRADE • AAP • APTA • ASPEN • ACEP • ASCO • AUA	-	-
	Controlled natural language	**-**	**-**	For authoring recommendation.	**-**	**-**
	Deontic terminology	**-**	**-**	Automatically is suggested by the tool based on equilibrium or the preponderance of benefits, risks, harms and costs, and evidence quality.	**-**	**-**
	Voting	By the integration with iEtD from all PICOs.	Only in the process of formulating guideline questions	-	-	Can be customized for different purposes
Document management	Change control (tracking)	Not for the full guideline. Only on text	**-**	**-**	**-**	Not for the full guideline. Only on: Text, comments, Files
	Managing versions (version control)	**-**	**-**	**-**	**-**	For content type “text”
	Template-based authoring	Template for some sections of a guideline.	Flexible template in the embedded text editor including subject-headings	For developing recommendations.	**-**	**-**
	Guideline repository management	Online database	Online database	No Database. The file should be stored by users manually on their own PC.	Online database	Online database
Guideline content enhancement	Tagging	• ICD-10 • SNOMED-CT • ATC • RxNorm • MeSH • ICPC-2 • LOINC • UMLS • NEKLAB (Norwegian lab coding)	**-**	**-**	• MeSH	**-**
	EHR linking	• Open EHR [70]	**-**	**-**	**-**	**-**
Import, export and publication	Import file formats	References: • Manually • Import from PubMed, • Import RIS file	SoF: • RevMan5	**-**	Attach to guidelines: • PDF file	Bibliographic data including abstract: • XML
		SoF: • RevMan5 (import as a reference)				• MS Word • PDF
	Export file formats	Guideline: • PDF • JSON	Recommendation: • MS Word • PDF • HTML	Recommendation • MS Word	Guideline: • PDF	Bibliographic: • Medline and EndNote generated XML
		Evidence profile and PICO: • JSON • RevMan5 • MS Word • MAGIC file format	SoF: • RevMan5 • PDF • MS Word • HTML			
	Publishing	Web and specific apps for phone and tablets with offline access	• Web • Mobile	**-**	• Web	• Web
User experience enhancement	Wizard-based authoring	-	Only for • COIs management • Voting process	Only for • Developing recommendation	-	-
	Walkthrough user guide	-	For the main implemented features	-	-	-
	User manual	• Yes	• Yes	• Yes	-	• Yes

As shown in Table 3, four out of the five tools had common functionalities or features: collaboration, access control, guideline repository management, electronic publishing. The GRADE methodology is supported in three of the reviewed tools for rating of guideline recommendations. We found only two tools supporting MeSH annotation. We also identified that monitoring progress, reference management, managing versions (version control) and Change control (tracking) had least coverage. Results show that the identified themes and their features not only cover required functionalities and features for a GDT to support the guideline development process, but also include functionalities and features to improve guideline dissemination, guideline search, and integration of guideline recommendations to EHR. We note that we contacted the creators of the tools to confirm the results and our judgment.

## Discussion

In this section we provide an analysis of the results with suggestions for improvement.

1. *Conflict of Interest and user access*
  Although GRADEpro and Internet portal can manage conflict of interest (COI) and have role based user access, they lack fine-grained edit access for COIs on specific guideline sections. *We recommend fine-grained access control and administrator roles on section/recommendation level* with different restriction level.
2. *Workflow support*
  The ‘development checklist’ and ‘setting milestones and deadline’ in MAGICapp are not integrated with workflow management. Similarly, GRADEpro does not track task completion based on the milestone manager. *We recommend that GDTs interface with workflow management systems that can support the entire GL development process for distributed teams.* In order to control and track how a guideline under development moves from one team member to another based on their contribution and track the steps, proper workflow management system support is necessary. A workflow model to manage content from initial draft to the publishable form would be an improvement. *Plans milestones, checklists, progress of voting, review/approval should be considered. Progress monitoring at individual, group and project level is critically important. In addition, a workflow model should provide details about development/update, content changes, versions, feedback, task assignment, and active reminders. A customizable* workflow management system can be used to tailor development to local practice.
3. *Collaborative literature search and evidence analysis*
  Evidence assessment is a collaborative process and authors may need to highlight or annotate the reviewed article. Our results show that only Internet Portal can import articles to a repository accessible to team members for further analysis, data extraction with the aim of making a systematic review. *We recommend that GDTs supports annotation, annotation comments and storage of search results for each individual article.*
4. *Automatic tracking of changes and version control*
  Developing guidelines is a collaborative process and change tracking is intertwined with development process and conflict resolution. *We recommend that edit history, changes, forking, roll-backs and supporting user/role/group on the same granularity as COI-management and voting*. Furthermore, *we recommend that versioning may be automatic and milestone- or interval-based so as to simplify use of long-term, continuous development processes.*
5. *Customizable guideline template*
*We recommend that the GDT support use and creation of guideline templates.* This may significantly lower effort for developing new or local versions of a GL, and it will help less experienced developer teams. A customizable template can be used to tailor development to local practice and potentially increase tool adoption
6. *Usability of guideline development tools*
  Usability is a core concern for any software tool. BRIDGE-Wiz has been evaluated [7], but we did find few specific results for GDT usability. That is not surprising, since software engineering considers usability part of practice, and not research*. However, we recommend that user interface design, wizard-based and template-based authoring, interactive user guides, and workflow management support be thoroughly evaluated with respect to utility and usability.*
7. *Electronic GL publication and usability*
  In our previous studies about usability evaluation of GLs on the Web [40–43] we found that presentation format, layout, navigation and search functionality were important [44]. GDTs affect publication format and functionality and thus guideline usability. For example, as MAGICapp and GRADEpro allow publishing on various platforms, which may be both a challenge and boon in practice. As another example, in our previous study we found that guideline users are concerned with credibility of recommendations; hence providing additional information with the graded recommendation would increase their confidence in choosing recommendations [43]. We recommend that GDTs provide or enable easy publication of references, author justification as well as evidence in a comprehensive, yet unobtrusive way.
8. *Guideline annotation*
  EHR integration is very important, but relies on computational formalization, and domain modelling, which begins where we stopped. However, simple GL annotation may enhance search and retrieval related to specific patients. MAGICapp describes EHR content in the GL, enabling the GL recommendations to be instantiated with current patient data.

The analysed GDTs in this paper are developed by a joint collaboration of multidisciplinary and international teams (MAGICapp (Norway, Canada, Spain, Germany, Scotland, Lebanon, and USA), GRADEPro (Canada, Chile, Brazil, USA, American University of Beirut, Spain, Italy, Germany, Norway, USA, UK, World Health Organization (Switzerland), Finland, and Denmark), BRIDGE-Wiz (USA), internet Portal (Germany), and Håndboka (Norway)), hence the presented results and findings will be applicable to other guideline development organisations in other countries.

Much research and development have gone into formalizing GLs to make them computer interpretable. The accompanying software development tools, or integrated development environments [45] support the encoding of guidelines to facilitate content-based search, integration of electronic health records with guidelines and most importantly, executable decision and process support. [46]. As already stated, we regard this as a later step of GL deployment and implementation similar to software development, and not relevant for the initial GL authoring. Future work should explore the gaps authoring guideline content and for making them computer-interpretable. This will ease the flow of evidence to the clinic. The electronic development of clinical guidelines can facilitate the formalization step if they can communicate in terms of language, syntax, and semantic provided by the tools; therefore it reduces the challenges of the guideline maintenance process and therefore formalization process.

## Conclusion

In this study we searched literature and contacted guideline development organisations to identify tools and CMS for development of GLs. We reviewed five academically reported GDTs (MAGICapp, GRADEpro, BRIDGE-Wiz, and Internet Portal) and Håndboka, as a representative of GL-specific CMSs. The results in this paper are useful for organisations and authors assessing and selecting a GDT.

By reading tool manuals, exploring the tools hands-on, comparing them against other, we uncovered, and proposed 8 themes that represent tool functionalities and features. Tools were selected according to their support of the guideline development process. Finally, the tools were characterized according to the 8 themes. Our purpose was not an overall GDT ranking, since GDTs are made for different purposes (i.e. only developing recommendations or a full guideline) and methodologies (i.e. developing recommendations based on GRADE). The identified themes can however be used for comparing tools, and in the future reconcile common features, and components. As guideline development manuals and methods are becoming more aligned, even standardized, this will drive alignment of GDTs. Furthermore, guideline representation and language standards encourage vendors to develop commercial applications for guideline implementations and EHR integration.

The tools may have limitations, and features, that are only possible to uncover after realistic use. Guideline author and editor requirements are also best captured when actually using the GDTs. Further study is required to understand if, and how, standard terminologies and ontologies impact the guideline encoding process. So, in summary:

What was already known?

- ▪ Guideline authors mainly use text editors or CMS for authoring and publishing clinical guidelines.
- ▪ The need for software support of GL authoring, maintenance and dissemination.

What this study added to our knowledge:

- ▪ Identification of research prototypes and tools from literature, meetings, professional networks and health organizations.
- ▪ Functionalities and features of representative GDTs.
- ▪ Eight themes representing the GDT features.
- ▪ Similarities and differences with regard to features among reviewed GDTs
- ▪ The GDT application area and supported development methodology.

## Additional file

Additional file 1: Details of the tools analysis. The additional file elaborated in details, when necessary, on the analysis of the reviewed tools (MAGICapp, GRADEpro, BRIDGE-Wiz, Håndboka, and Internet Portal) according to the presented results in Table 3. Each bold title represents the theme according to the uncovered themes in Table 2. (PDF 187 kb)

## Abbreviations

CMS: Content management systems
GDT: Guideline development tool
G-I-N: Guidelines International Network
GLs: Clinical guidelines
Internet Portal: Internet Portal for guideline development
