# Supplementary material for: Reviewing clinical guideline development tools: features and characteristics
Source: BMC Med Inform Decis Mak. 2017 Sep 4;17:132. doi: 10.1186/s12911-017-0530-5 (PMC5584508; doi:10.1186/s12911-017-0530-5)
Supplement: Additional file 1: — Details of the tools analysis. The additional file elaborated in details, when necessary, on the analysis of the reviewed tools (MAGICapp, GRADEpro, BRIDGE-Wiz, Håndboka, and Internet Portal) according to the presented results in Table 3. Each bold title represents the theme according to the uncovered themes in Table 2. (PDF 187 kb) [file 12911_2017_530_MOESM1_ESM.pdf]

Team and contribution management

- *Conflict* of interest

GRADEpro supports COI management based on four different forms that are developed by different organisations: ICMJE [4], WHO [5], NHF (McMaster-NHF Guideline Panel conflict of interest form), and MSPSC [6]. The moderator can choose only one type of form for each guideline under development and send notification to the team members to fill the COI form. The moderator can access the results and able to send a reminder to the members in case they are delayed in filling out the COI form.

The coordinator in Internet Portal can invite the members to declare their conflict of interests. The coordinator can monitor if the COI forms are completed and send reminders to the members who have not filled out the form. In addition, the coordinator has the right to view and make a decision about the COI results: 'no or insignificant COI', 're-evaluate COI', 'statement is incomplete or unclear and reworking is necessary'. Coordinators can add their comments to the COI decisions.

Project management

- *Development checklist*

As is presented in Table 3, MAGICapp has a guideline development checklist in three categories: 'planning', 'content development', 'publication, evaluation and updating'. The designed checklist follows the formats suggested by Guideline International Network [1], AGREE [2], and GIN-McMaster Guideline Development Checklist [3]. The provided checklist combines all the items from the above mentioned sources. Authors or mediators should manually insert the information into the checklist based on their progress in the guideline development.

- *Milestone and deadlines*

Each milestone can include information such as title and additional comments. A detail of information that each tool supports to include in each milestone is presented in Table 3. All the set milestones can be added to the embedded calendar and authors can have a comprehensive view of the upcoming events and milestones. In Internet Portal, authors can set the milestones and deadlines to the standard calendar format 'iCal' and 'vCal', and store it in their local calendar such as MS Outlook.

- *Progress monitoring*

In Internet Portal, authors can monitor the progress of voting (completed, not completed) and surveys (percentage of the completion). For every question that is sent for voting (voting status), a colour-coded notice indicates the completion status to simplify the identification of open questions. A statistical analysis of results simplifies online voting and survey considerably, as it presents the total number of recorded votes and answered surveys, the percentage and absolute answer distribution. In addition, activity logs based on date, time, and the contributor name can be monitored by the users.

In MAGICapp, authors can set manually the progress of 'evidence profiles' to: 'Under development', 'For review', 'Under updated', and 'Finished for publication'. On all PICOs, authors can get a summary of these labels on the top level. Each of this status is associated to a colour code. In addition, guidelines as a whole have the status settings: «Under development», «For Internal review», «For External review», «Finished for publication», «Under update», «Updated» and «Blank»(not set). However, these statuses are not integrated to 'guideline development checklist' to enable authors monitor the extent to which guideline has been completed.

Recommendations have colour labels for Strength (which gives you an idea of how far the process have come), but no status labels for Authors.

Evidence management

- *Search strategy and history*

MAGICapp provides links to six search databases: 1) Epistemonikos ("collaborative, multilingual database of research evidence and knowledge translation products that are usually referred as 'evidence', according to the meaning given in Evidence-Based Health Care" [7]), 2) SuperFilters (literature search service implemented by McMaster university), (3) PubMed, 4) International Guideline Library (search engine from guideline international network), 5) Dynamed (evidence-based clinical information resource), and 6) The Knowledge Egg (search service that has useful information about published guidelines, protocols, repositories and available search databases). Authors can insert a search keyword in the designated search-boxes and the 'search' buttons direct the user to the selected search engine chosen by the user (i.e. PubMed). We could not find any designated section for a literature search in MAGICapp where authors can document their search criteria and strategy. However, authors can use a manual reference as a search strategy and document their search by narrative text summary in the tab Abstract.

In the Internet Portal for guideline development, the search strategy can be documented in the portal along with the import history in a literature collection (i.e. when authors import the literature found from PubMed, they can create a folder under literature collection and document the search strategy.

- *Evidence repository*

As is presented in Table 3, in Internet Portal, the found literature can be imported to the repository including their bibliographic data (abstract, journal, authors, affiliation) and abstract. In addition, authors can attach the full text of articles to the imported bibliographic records. All the members have access to the imported literature, and can comment, manage and evaluate the imported literature. In MAGICapp, it is possible to attach a file from literature to an imported reference. However, it is not possible to have a literature repository for all the found literature.

- *Citation and references*

Even though MAGICapp supports adding citations from imported references, when author deletes or update the reference library, the annotated references are not updated automatically. Therefore, the author should manually update the cited referenced in the guidelines based on the references library.

Guideline development

- *Evidence assessment*

In the 'evidence profile' in MAGICapp, authors can add dichotomous outcome, continues outcomes and practical consequences and specify their confidence in the effect estimates (quality assessment) and the type of study that the evidence is based on. GRADEpro supports GRADE methodology for assessing evidence in 'evidence table'. In the Internet Portal, authors can assess the evidences based on Scottish Intercollegiate Guidelines Network (Sign) [8] and Oxford [9]. In addition, they can make customized forms for evidence assessment (literature appraisal) based on different organizations' needs. The Sign, Oxford and the customized forms are integrated into the portal. The current version of portal does not support GRADE methodology.

- *Quality rating*

MAGICapp supports grading recommendations by providing a colour code with four provided options. The four colours codes are: grey (not set), yellow (weak recommendation), green (strong recommendation), and blue (practice statement). GRADEpro also provides a colour code for grading of recommendations. However, assigning the colour codes is generated by the system based on the 'balance of consequences' and 'type of

recommendation' that the author specifies in the designated template. BRIDGE-Wiz supports rating of recommendations based on different methods: GRADE methodology (Grades of Recommendation Assessment, Development and Evaluation), the systems in use by the American Academy of Paediatrics (AAP), American College of Emergency Physicians (ACEP), American Society of Clinical Oncology (ASCO), American Urological Association (AUA), American Society for Parenteral and Enteral Nutrition (ASPEN), and American Physical Therapy Association (APTA) [10-12]. Therefore, different versions of BRIDGE-Wiz are available based on the supported methodology for rating of the recommendations. The version that is based on GRADE calculates strengths of recommendations based on the author's input (the balance between the benefits and harm, and identifying evidence quality). The strength of recommendations is presented to the author accompanied by a table that presents its details. The strength of recommendations in the output file (MS Word) is highlighted by bold text rather than a colour code. BRIDGE-wiz does not support an evidence table (summary of finding table) in the process of authoring recommendations. However, authors can state evidence quality in the designated template as high, moderate, low, and very low.

- *Deontic terminology (Terminology and language)*

In BRIDGE-Wiz, the deontic terminology is automatically suggested based on equilibrium or the preponderance of benefits, risks, harms and costs, and evidence quality.

- *Voting*

Support of voting to achieve consensus: GRADEpro supports voting only in the process of identifying 'management' and 'diagnostic' questions that have to be addressed in the guidelines. The steps of voting to achieve consensus are: brainstorming, completed list, prioritising, final list for approval, approved list, and the last step 'finished'. When the moderator sends question(s) for brainstorming, the group members receive an email with a link provided to participate in the brainstorming process. Group members are able to add more questions (management or diagnostic) to the list that is initiated by moderator. The 'completed list' of questions can be sent to group members via email by moderator for prioritising (prioritising the importance of the questions that have to be addressed in the guideline under development). A list of the questions with their median ratings (it is calculated by the system based on priorities specified by team members) are presented to moderator in the 'final list for approval'. However, the moderator has the right to override the ratings. The questions can be categorized in three main categories based on the ratings: 'questions to be answered with recommendations', 'questions to be listed as potentially important but not to be addressed in this document',

‘questions to be excluded owing to at best limited priority or relevance for this document’. Each group member who receives the email with the provided link can state his/her agreement or disagreement with the prioritised questions. In addition, he/she can add comments about his/her disagreement in the provided link enclosed in the email. The moderator can close the voting process in this step and the approved questions are considered for ‘quality assessment’ and ‘summary of findings’ table while the remaining questions are added to the ‘appendices’ section of the guideline document.

In MAGICapp it is possible to support voting to achieve consensus in evidence assessment by the integration with iEtD from all PICOs (ietd.epistemonikos.org)

In Internet Portal, the coordinator can initiate voting. The voting feature can be customised for different purposes (i.e. identifying key questions for the guideline or for development of recommendations). Authors can add comments to their vote. In addition, voting can be used as a tele voting system in the context of consensus conferences and meetings. The votes casted can be monitored by the coordinator, and the final report is accessible to the team members after termination of the vote. The results of voting are anonymous. The portal enables coordinator to design non-repeated surveys or repeated voting in the sense of the Delphi method.

Document management

- *Change control*

In MAGICapp, authors are able to see the guideline update information including main editor, last edit date, first published date, and last offline download by the users; However, it is not possible to track all of the updates for all sections of the guidelines. Tracking and approving changes are possible only in the integrated text editors that are designated to insert text (i.e. background information, abstract). In the designated text-editors, if the author activates ‘start tracking changes’, then the system highlights the sentences in colour (including the author’s name) that are added or deleted based on the collaborators’ contribution. Therefore, the other contributors can track changes and accept or reject updates. We could not find tracking of changes on the sections such as content of evidence table, references, and section names.

Similarly, Internet Portal can track modifications in an integrated text editor by highlighting them in colour. In addition, authors can quickly identify altered files, texts, discussions (comments) in the ‘status section’ of the guideline in the portal. In the ‘status section’, a quick overview of new or changed content with a link to its guidelines sectors is presented. The new updates are illustrated in the form of a list including the following information: the title of the relevant content that is linked to the content, the author of the content, the date and

time of creation or modification, the location of the content within the guidelines in the form of a path by individual path elements.

The GRADEpro, BRIDGE-Wiz and Håndboka do not present track of updates for a guideline that is under development. By contacting to developer team, we found that MAGICapp, GRADEpro, Håndboka can keep track of all changes that have been made to the guideline in their database (log of changes). However, we do not know if the logs of changes are stored in the database based on users' account, and if the metadata associated with the guidelines is tracked, as we did not have access to the database to evaluate ourselves. Our results in this matter are based on the feedback we received from the tools' support team.

- *Managing versions (version control)*

Our review shows that versioning functionality is not implemented in any of the reviewed tools except Internet Portal. Versioning is only available for content type 'text' and changes are automatically versioned. This means that it stores all change statuses of online texts throughout the processing period. It is also possible to view and make changes where appropriate to reverse the updates. Authors are able to view a list of the current and all previous versions, indicating the continuous version number, the author, creation date and the comments. Furthermore, it is also possible to compare the current version or the previous versions with the other versions. The new parts of the text are highlighted in green and deleted text in red. The group leader and coordinators have the right to reverse changes.

- *Template-based authoring*

The MAGICapp guideline template includes key subjects such as background text, recommendations, effect estimates, key information, rationale, practical advice, adaptation, references and discussion section (which enable the authors to comment on a guideline). The GRADEpro guideline template includes guideline title, authors, disclosure of potential conflicts of interest, review group, table of contents, executive summary, introduction, methodology, how to use these guidelines, key questions, recommendations, introduction and background, scope, methods, group composition, group meetings, and appendices. We note that in GRADEpro, the template is provided in a web-based text editor and the subject headings are editable. The template in BRIDGE-Wiz includes date, key action statement, action, aggregate evidence quality, benefits, risk, harm, cost, value judgments, intentional vagueness, role of patient preferences, exclusions and notes. However, BRIDGE-Wiz does not have a template for a full guideline. In Håndboka, it is the author's decision or preference to include key subject headings a web-based text editor. In Internet Portal authors can develop the full guideline in

the embedded text editor and decide about the guideline template. This means that the Internet Portal does not have any specific template for the guideline.

Guideline content enhancement

- *Tagging*

MAGICapp enables users to tag the standard codes from ICD-10 (version 10) [13], SNOMED-CT [14], ATC [15] and RxNorm [16], MeSH [17], and ICPC-2 [18] to PICO (patient, intervention, comparator and outcome) questions. The ontologies and terminologies MAGICapp supports include: ICD-10 (version 10) [13], SNOMED-CT [14] (for drug groups), LOINC [19], ATC [15], RxNorm [16], UMLS [21] and MeSH [17] for diseases (diagnose).

In Håndboka, each guideline can be tagged to MeSH terms that ease searching published guidelines.

- *EHR linking*

In MAGICapp, each recommendation can be assigned to EMR elements (up to 10 elements) from multiple ontologies. The EMR elements that MAGICapp supports are classified into four categories: laboratory tests, observation/measurements, drug groups, and registered diseases from open EHR [20] (for laboratory tests and observation/measurements).

Import, export and publication

- *Import file formats*

In MAGICapp, references can be added to the guideline in three different ways: 1) manually (it has a template to add reference details), 2) import from PubMed (by adding PubMedID), 3) upload references using RIS file (is a standardised tag format which enables citation programmes to exchange data; it is supported by a number of reference managers such as digital libraries, IEEE Xplore, Scopus, the ACM Portal, Scopemed, ScienceDirect, and SpringerLink). Summary of finding (SoF) can be imported in RevMan5 file format as an attachment to the references. RevMan5 is the file format of the Cochrane Collaboration’s software for preparing and maintaining Cochrane reviews. Furthermore, additional files can be added to the reference PDF, picture or a document format.

In GRADEpro, it is possible to import a GRADEpro file format from a previously developed project or RevMan5. In Håndboka authors can upload a PDF file to the guidelines as an attachment. In Internet Portal, the bibliographic data including abstract can be imported in XML format. It is also possible to upload files in MS Word and PDF file into the portal.

- *Export file formats*

MAGICapp exports the guideline content to PDF and JavaScript Object Notation (JSON). It also exports the PICOs (patient, intervention, comparator and outcome) including evidence profile to RevMan5, JSON, MS Word, or their own format in MAGICapp. In GRADEpro users can export the SoF table to RevMan5 format, HTML, PDF, and MS Word. Recommendations can be exported to MS Word, PDF and HTML. The export format of recommendations in BRIDGE-Wiz is MS Word. The guidelines authored in Håndboka can be exported only to PDF file format. In Internet Portal it is possible to export bibliographic data in Medline and Endnote-generated XML. In general, all uploaded files can be downloaded from the portal.

- *Publishing medium (Electronic Publishing)*

The published guidelines in MAGICapp can be used in off-line mode, so if the user does not have access to the internet, the off-line version of the guideline can be accessible. GRADEpro enables authors to preview the guideline format on mobile devices before publishing. In addition, if an author wants to preview the guideline on his/her phone, it is possible to receive the mobile version on the phone by scanning the generated QR code by GRADEpro.

Others (User enhancement

- *Wizard-based authoring*

BRIDGE-Wiz has a wizard that presents the sequences of steps to develop a 'key action statement profile' (recommendations). Authors can develop recommendations by following up the wizard that specifies: what (the type of activity such as examining, prescribing, and documenting), action statement, when (the condition that will be performed), benefits, risks, harms, equilibrium and preponderance of benefits, risks, and harms, and evidence quality (high, moderate, low, and very low). The author is able to go forward and backward to edit recommendations before exporting the final output file. GRADEpro only has a wizard for the process of managing COIs and voting. We note that when each step in the wizard has completed it is not possible to move backward to edit the information in the previous steps.

References

1. Qaseem A, Forland F, Macbeth F, OllenschlÃĪger G, Phillips S, van der Wees P. Guidelines International Network: toward international standards for clinical practice guidelines. Annals of Internal medicine. 2012;156(7):525-31.
2. Terrace L. Development and validation of an international appraisal instrument for assessing the quality of clinical practice guidelines: the AGREE project. Qual Saf Health Care. 2003;12:18-23.
3. Guideline Development Checklist. 2014. http://cebgrade.mcmaster.ca/guidelinechecklistonline.html. Accessed 22 July 2015 2015.
4. Editors ICoMJ. Conflict of Interest Disclosure Forms. 2015 http://www.icmje.org/about-icmje/faqs/conflict-of-interest-disclosure-forms/. Accessed 22 July 2015 2015.
5. World-Health-Organization. WHO Handbook for Guideline Development Process - World Health Organization. 2010. http://www.who.int/hiv/topics/mtct/grc_handbook_mar2010_1.pdf. Accessed 10.03.2015 2015.
6. Schünemann H, Mustafa R, Brozek J, Carrasco-Labra A, Brignardello-Petersen R, Wiecioch W. Saudi Arabian Manual for Healthcare Guideline Development.
7. About Epistemonikos. http://www.epistemonikos.org/en/about_us/who_we_are. Accessed 27 July 2015 2015.
8. SIGN. Scottish Intercollegiate Guidelines Network Methodology checklist. SIGN. 2014. Available from: http://www.sign.ac.uk/methodology/checklists.html.
9. Oxford. Oxford Centre for Evidence-Based Medicine. Levels of evidence. http://www.cebm.net/oxford-centre-evidence-based-medicine-levels-evidence-march-2009/ Accessed May 2016.
10. Shiffman RN, Michel G, Rosenfeld RM, Davidson C. Building better guidelines with BRIDGE-Wiz: development and evaluation of a software assistant to promote clarity, transparency, and implementability. Journal of the American Medical Informatics Association. 2012;19(1):94-101. doi:10.1136/amiajnl-2011-000172.
11. West SL, King V, Carey TS, Lohr KN, McKoy N, Sutton SF et al. Systems to rate the strength of scientific evidence. Agency for Healthcare Research and Quality, US Department of Health and Human Services; 2002.
12. Yale0University. Available Versions of BridgeWiz. 2011. http://gem.med.yale.edu/BRIDGE-Wiz/. Accessed May 2016.
13. Organization WH. International statistical classification of diseases and health related problems (The) ICD-10: World Health Organization; 2004.
14. Donnelly K. SNOMED-CT: The advanced terminology and coding system for eHealth. Studies in health technology and informatics. 2006;121:279.
15. Miller G, Britt H. A new drug classification for computer systems: the ATC extension code. International journal of bio-medical computing. 1995;40(2):121-4.
16. Liu S, Ma W, Moore R, Ganesan V, Nelson S. RxNorm: prescription for electronic drug information exchange. IT professional. 2005;7(5):17-23.
17. Nelson SJ, Johnston WD, Humphreys BL. Relationships in medical subject headings (MeSH). Relationships in the Organization of Knowledge. Springer; 2001. p. 171-84.
18. Lamberts H, Okkes I. ICPC-2, International Classification of Primary Care. 1998.
19. McDonald CJ, Huff SM, Suico JG, Hill G, Leavelle D, Aller R et al. LOINC, a universal standard for identifying laboratory observations: a 5-year update. Clinical chemistry. 2003;49(4):624-33.
20. Beale T, Heard S, Kalra D, Lloyd D. OpenEHR architecture overview. The OpenEHR Foundation. 2006.
21. Lindberg DA, Humphreys BL, McCray AT. The Unified Medical Language System. Methods of information in medicine. 1993;32(4):281-91.
